# Asiatic Acid Attenuates Osteoporotic Bone Loss in Ovariectomized Mice Through Inhibiting NF-kappaB/MAPK/ Protein Kinase B Signaling Pathway

**DOI:** 10.3389/fphar.2022.829741

**Published:** 2022-02-08

**Authors:** Mingming Dong, Jican Zeng, Chenyu Yang, Yisen Qiu, Xinjia Wang

**Affiliations:** ^1^ Department of Spine Surgery, The Second Affiliated Hospital, Shantou University Medical College, Shantou, China; ^2^ Department of Orthopedic, Affiliated Cancer Hospital, Shantou University Medical College, Shantou, China

**Keywords:** asiatic acid, protein kinase B, osteoclastogenesis, osteoblast, osteoporosis

## Abstract

Osteoporosis is a condition associated with osteolytic bone disease that is primarily characterized by inordinate osteoclast activation. Protein kinase B (Akt) pathways activated by receptor activator of nuclear factor kappa-B ligand (RANKL) are essential for osteoclastogenesis. Asiatic acid (AA) is a natural pentacyclic triterpenoid compound extracted from a traditional Chinese herb that exhibits a wide range of biological activities. AA has been found to alleviate the hypertrophic and fibrotic phenotype of chondrocytes via the Akt signaling pathway. In this study, we investigated whether AA alleviated bone loss by inhibiting the Akt signaling pathway during osteoclastogenesis and its effect on osteoblasts. The effect of AA cytotoxicity on mouse bone marrow-derived macrophages/monocytes (BMMs) was evaluated *in vitro* using a Cell Counting Kit-8 assay. The effects of AA on osteoclast differentiation and function were detected using tartrate-resistant acid phosphatase (TRAP) staining and a pit formation assay. A Western blot and qRT-PCR were conducted to evaluate the expression of osteoclast-specific genes and protein signaling molecules. In addition, alkaline phosphatase and alizarin red staining were performed to assess osteoblast differentiation and mineralization. The bone protective effect of AA was investigated *in vivo* using ovariectomized mice. we found that AA could dose-dependently inhibit RANKL-induced osteoclastogenesis. Moreover, the pit formation assay revealed that osteoclast function was suppressed by treatment with AA. Moreover, the expression of osteoclast-specific genes was found to be substantially decreased during osteoclastogenesis. Analysis of the molecular mechanisms showed that AA could inhibit NF-kappaB/MAPK/Akt signaling pathway, as well as the downstream factors of NFATc1 in the osteoclast signaling pathway activated by RANKL. However, AA did not significantly promote osteoblast differentiation and mineralization. The *in vivo* experiments suggested that AA could alleviate ovariectomy-induced bone loss in ovariectomized mice. Our results demonstrate that AA can inhibit osteoclastogenesis and prevent ovariectomy-induced bone loss by inhibiting the NF-kappaB/MAPK/Akt signaling pathway. The discovery of the new molecular mechanism that AA inhibits osteoclastogenesis provides essential evidence to support the use of AA as a potential drug for the treatment of osteoclast-related diseases.

## Introduction

Osteoporosis is a metabolic bone disease with systemic bone degeneration characterized by reduced bone mass and destruction of the bone microstructure. Moreover, osteoporosis is associated with complications of bone deformities and the brittle fractures caused by osteoporosis are often catastrophic for middle-aged and elderly individuals (Anne et al., 2001; Sattui and Saag, 2014). Bone remodeling is a process that exists in a dynamic equilibrium, and its stability is primarily dependent on the regulation of osteogenic bone formation and osteoclastic bone resorption (Zaidi, 2007). Thus, a disruption in bone homeostasis caused by over-activation of the bone resorption function of osteoclasts is an important cause of osteoporosis. Since osteoclasts are the main cells with a bone resorption function within the bone tissue, the inhibition of osteoclasts may represent the primary target for the treatment of osteoporosis (Schell et al., 2006; Novack and Teitelbaum, 2008).

Osteoclasts derived from bone marrow hematopoietic stem cells are multinucleated giant cells formed by the fusion of multiple mononuclear macrophages (Yahara et al., 2020). It has now been well-established that both macrophage colony-stimulating factor (MCSF) and nuclear factor kappa-B ligand (RANKL) are two necessary cytokines responsible for osteoclast formation (Boyce, 2013). After binding to RANKL, the receptor activator of nuclear factor kappa-B (RANK) expressed on the surface of osteoclast precursor cells can recruit and activate the adaptor protein tumor necrosis factor receptor-associated factor 6 (TRAF6). TRAF6 then regulates osteoclast bone resorption and differentiation through activated downstream signal transduction cascades (e.g., NF-κB/MAPK/Akt signaling pathway) (Boyle et al., 2003; Novack, 2011). The extracellular signal-regulated kinase (ERK) signaling pathway is involved in the regulation of osteoclast differentiation, migration, and bone resorption (He et al., 2011). Osteoclast fusion is dependent on c-Jun N-terminal kinase (JNK) signaling (Chang et al., 2008). In addition, the p38 signal transduction pathway both stimulates osteoclast formation and maturation and plays an irreplaceable role in the osteoclast and osteoblast coupling process (Cong et al., 2017). Akt participates in osteoclast formation primarily by regulating the glycogen synthase kinase 3*β* (GSK3*β*)/nuclear factor of activated T cells cytoplasmic 1 (NFATc1) signaling pathway (Moon et al., 2012). NFATc1 is a vital transcription factor that regulates the expression of osteoclast specific genes [e.g., cathepsin K (CTSK) and dendritic cell-specific transmembrane protein (DC-stamp)] (Takayanagi et al., 2002; Kim et al., 2008; Balkan et al., 2009), ultimately impacting the formation of multinucleated mature osteoclasts.

At present, an increasing number of natural plants have achieved considerable results for the treatment of a wide variety of clinical diseases. For instance, natural products that can protect against osteoporosis has received increased recognition (Guo et al., 2021; Liu et al., 2021; Zhang et al., 2021). Asiatic acid is a pentacyclic triterpenoid compound from *Centella asiatica*. It is currently thought that asiatic acid is associated with anti-oxidative stress reactions, anti-inflammatory responses, the protection of nerve cells, as well as the anti-tumor effect (Wu et al., 2017b; Wang et al., 2018; Yuyun et al., 2018; Chen et al., 2019). Additionally, multiple studies have shown that there was a substantial correlation between the pharmacological effects of asiatic acid and the Akt signaling pathway (Ren et al., 2016; Hao et al., 2018; Gou et al., 2020), including the biological basis of asiatic acid in the degradation of articular cartilage treatment (Liu et al., 2020). Recently, asiatic acid has been shown to effectively inhibit osteoclast differentiation and alleviate osteoporosis through the TGF-*β*/NF-κB/NFATc1 pathways (Huang et al., 2019; Hong et al., 2020). However, the mechanism by which asiatic acid affects bone metabolism has not been thoroughly elucidated to date. Moreover, whether asiatic acid plays a role in anti-osteoporosis by inhibiting Akt pathway during osteoclastogenesis remains unknown. Additionally, the effect of asiatic acid on osteoblasts has not been shown. Thus, in this study, we investigated the possible novel molecular mechanism by which asiatic acid affects osteoclasts and its corresponding effect on osteogenesis. The effect of asiatic acid on bone protection was further explored *in vivo*.

## Materials and Methods

### Reagents

Asiatic acid was purchased from Selleck Chemicals. Alpha modification of Eagle’s medium (*α*-MEM), fetal bovine serum (FBS), streptomycin, and penicillin were purchased from Gibco (Thermo Fisher Scientific, Waltham, MA, United States), and RANKL and M-CSF were purchased by R&D Systems (Minneapolis, MN, United States). The internal reference was detected using a mouse-derived *ß*-Actin antibody (ProteinTech, Rosemont, IL, United States). Rabbit-derived antibodies specific for NF-κB p65, p-NF-κB p65, IκB*α*, Phospho-Akt (Thr308), Akt, p38, phospho (p)-p38, ERK, p-ERK, JNK, and p-JNK were provided by Cell Signaling Technology (CST, Danvers, MA, United States). Mouse-derived antibodies specific for NFATc1 was provided by Santa Cruz Biotechnology (Dallas, TX, United States). The anti-rabbit or anti-mouse horseradish peroxidase-labeled secondary antibodies were purchased from Beyotime (Beyotime, Shanghai, China).

### Cell Culture

Mouse bone marrow-derived macrophages/monocyte (BMMs) were extracted from the tibia and femoral bone marrow cavities of 5 week-old C57BL/6J mice. BMMs were cultured in *a*-MEM medium (1% penicillin/streptomycin, 10% FBS, and 30 ng/mL M-CSF) for 72 h in a cell incubator at 37°C and 5% CO_2_. BMMs were grown in a T75 culture flask with a diamond-shaped attachment and used in subsequent experiments.

### Cell Counting Kit-8

BMMs were inoculated into 96-well plates at 1 × 10^4^/well. After 12 h of adherence, the cells were treated with different concentrations of asiatic acid (0, 10 μM, 20, 40, 80, and 160 μM) for 48 h. Next, 10 μL CCK-8 reagent (Dojindo, Gaithersburg, MD) was added to each well and incubated at 37°C for 2 h under light. A microplate reader (Tecan, Mannedorf, Switzerland) was used to measure the absorbance of each well at 450 nm.

### Tartrate-Resistant Acid Phosphatase Staining

BMMs (1 × 10^4^ cells) were induced by *a*-MEM containing 30 ng/mL M-CSF and 50 ng/ml RANKL with different concentrations of asiatic acid (0, 20 μM, 40, and 80 μM). Changes in cell morphology were observed every 48 h and each group was replaced with the above-mentioned medium containing different concentrations of asiatic acid. After about 7 days, the control group differentiated into vacuolar-fused osteoclasts (≥to three nuclei). The cells were fixed with 4% paraformaldehyde and stained with Tartrate-resistant acid phosphatase. The osteoclasts were photographed under a light microscope (ZEISS, Germany). The number and area proportion of TRAP-positive osteoclasts (≥3 nuclei) were counted. ImageJ software (NIH, Bethesda, MD, United States) was employed to analyze the area proportion of TRAP-positive osteoclasts (≥3 nuclei).

### Pit Formation Assay

The pit formation assay was conducted in 96-well hydroxyapatite plates (OsteoAssay Surface plates; Corning, NY, United States). First, the BMMs were cultured and differentiated for 5 days until the initial formation of osteoclasts, then the initially formed osteoclasts were digested and seeded into 96-well bone plates at a cell density of 1 × 10^4^/well. Overnight, the initial formation of osteoclast cells were treated with various concentrations of asiatic acid (0, 20, 40, and 80 μM). The culture medium was replaced with fresh medium every 48 h. Approximately 5 days later, the cells were treated with 5% sodium hypochlorite for 5 min, rinsed in pure water, and the absorbable area of the osteoclasts was photographed under a light microscope (ZEISS, Germany). ImageJ software (NIH, Bethesda, MD, United States) was employed to analyze the resorbed area for each well.

### Polymerase Chain Reaction Analysis

BMMs were inoculated into a 6-well plates at a density of 2 × 10^5^/well. BMMs were induced by 30 ng/mL M-CSF and 50 ng/ml RANKL for 7 days with or without asiatic acid. We used RNAiso Plus (TaKaRa Biotechnology, Otsu, Japan) to extract the total RNA from the cells in accordance with the manufacturer’s instructions. cDNA was generated from 1 μg RNA using a reverse transcription kit (TaKaRa Biotechnology, Otsu, Japan). A TB Green Premix Ex Taq kit (TaKaRa Biotechnology, Otsu, Japan) was used to determine the level of gene expression. The primers were list in Table1.

**TABLE 1:** The following primer sequences were used in the present study.

Gene	Forward primer	Reverse primer
NFATc-1	CCG​TTG​CTT​CCA​GAA​AAT​AAC​A	TGT​GGG​ATG​TGA​ACT​CGG​AA
CTSK	CTT​CCA​ATA​CGT​GCA​GCA​GA	TCT​TCA​GGG​CTT​TCT​CGT​TC
Acp5	CAC​TCC​CAC​CCT​GAG​ATT​TGT	CCC​CAG​AGA​CAT​GAT​GAA​GTC​A
c-Fos	CAC​ATT​GGG​GGT​AGG​AAC​AC	CCA​GTC​AAG​AGC​ATC​AGC​AA
V-ATPase-d2	AAG​CCT​TTG​TTT​GAC​GCT​GT	TTC​GAT​GCC​TCT​GTG​AGA​TG
DC-stamp	CAC​TCC​CAC​CCT​GAG​ATT​TGT	CCC​CAG​AGA​CAT​GAT​GAA​GTC​A
GAPDH	AGG​TCG​GTG​TGA​ACG​GAT​TTG	GGG​GTC​GTT​GAT​GGC​AAC​A

### Western Blotting

BMMs were seeded into six-well plates at a cell density of 4 × 10^5^/well. After permitted cellular attachment, the medium was changed to serum-free *a*-MEM medium and the BMMs were starved for 2 h. Asiatic acid (20 μM) was then added to the experimental group. After treatment with asiatic acid for 2 h, BMMs were stimulated by adding RANKL (50 ng/ml) for 0, 5, 10, 20, 30, and 60 min. On the other hand, BMMs were seeded into six-well plates at a cell density of 2 × 10^5^/well. BMMs were stimulated with RANKL for 0, 1, 3, and 5 days with or without asiatic acid. The cells were then lysed on ice to extract the total protein, and the protein concentration was measured using a bicinchoninic acid (BCA) protein concentration determination kit (Beyotime, Shanghai, China). Proteins of different molecular weights were separated on a 10% SDS-PAGE gel. The electrophoresed proteins were transferred to nitrocellulose membranes at 100 V for 90 min. The nitrate cellulose membranes were blocked with 5% skim milk at room temperature for 1 h and then incubated overnight at 4°C with the corresponding primary antibody. The membrane was washed three times with TBST, and secondary antibodies of the corresponding species were incubated at room temperature for 1 h. Images of target protein expression were obtained using a chemiluminescence imager (Bio-Rad, Hercules, CA, United States) with the help of extremely hypersensitive ECL luminescence reagent. The ratio of the gray value was measured by Image Lab (NIH, Bethesda, MD, United States).

### Alkaline Phosphatase and Alizarin Red Staining

Osteoblasts were isolated from the cranial bones of newborn SD rats. The cranial bone was cut into bone tissue masses of approximately 4–5 mm^2^ with tissue scissors, and bone tissue masses treated with 0.25% trypsin and 0.2% type Ⅱ collagen were cultured in a T75 cell culture flask containing 15 ml DMEM medium. The cells were seeded into 24-well cell culture plates at a cell density of 4 × 10^4^/well and cultured in an osteogenic induction medium (50 μg/ml vitamin C, 10 mM *ß*-glycerolphosphate, and 0.1 μM dexamethasone) with or without asiatic acid. The culture medium was changed every 3 days. Approximately 7 days after osteogenic induction, the cells were fixed with 4% paraformaldehyde and stained with alkaline phosphatase. Alizarin red staining was performed about 21 days post-osteogenic induction. In addition, it is important to avoid exposure to light during the staining process.

### Animal Experiments

The eight-week-old C57BL/6J mice were purchased from Beijing Weitonglihua Laboratory Animal Technology Co., Ltd. (Beijing, China). Animal experiments were carried out in the Animal Center of Shantou University Medical School (SUMC2019-406). This study was approved by the Ethics Committee of Medical Laboratory Animals of Shantou University Medical School. The mice were acclimated to the new environment for 1 week, and the mice were fed in a ventilated environment with a 12 h light and dark cycle. Adequate food and water up to standard was provided, and rooms are regularly disinfected by ultraviolet light. A total of 20 mice were randomly divided into four groups: 1) sham operation group (Sham); 2) ovariectomized group (OVX); 3) ovariectomized + estradiol group (OVX + E2); and 4) ovariectomized + asiatic acid group (OVX + AA). After the mice were anesthetized with 2% pentobarbital sodium, a midline incision was made on the sterilized back, and subcutaneous tissues were bluntly separated layer by layer. After opening the abdominal cavity, both ovaries were ligated using 3.0 mm sterile surgical sutures. In the sham operation group, only part of the subperitoneal fat was ligated. The status of the mice was closely observed for 1 week after the operation. The mice recovered 1 week after the operation, and were intraperitoneally injected with either asiatic acid (10 mg/kg), estradiol (0.15 mg/kg) or normal saline (NS) once every 2 days. Six weeks later, the left tibia of the mice was collected for micro-CT (SCANCO, Wangen-Brüttisellen, Switzerland) scanning to analyze the bone volume to the total volume. BV/TV), trabecular number (TB.N), and trabecular separation (TB.SP) among all groups. The scanned tibia were fixed with 4% paraformaldehyde for 24 h, and the tibia specimens were decalcified with 10% EDTA, dehydrated, embedded in paraffin, and sectioned. The bone tissue sections were stained with hematoxylin-eosin staining (HE) and tartrate-resistant acid phosphatase (TRAP), and the tibial bone microstructure of the mice was observed under a microscope to analyze the level of osteoporosis in the mice.

### Statistical Analysis

The experimental data provided in this study were expressed as the mean ± standard deviation (mean ± SD). Statistical analyses were performed using SPSS 23.0 (IBM, United States). Student’s t-test was used to evaluate the difference between two groups. A one-way analysis of variance (ANOVA) was used to compare the difference between above two groups. A *p*-value < .05 was considered to indicate that the differences were statistically significant.

## Results

### Asiatic Acid Inhibits Osteoclast Differentiation and Bone Resorption

**FIGURE 1:**
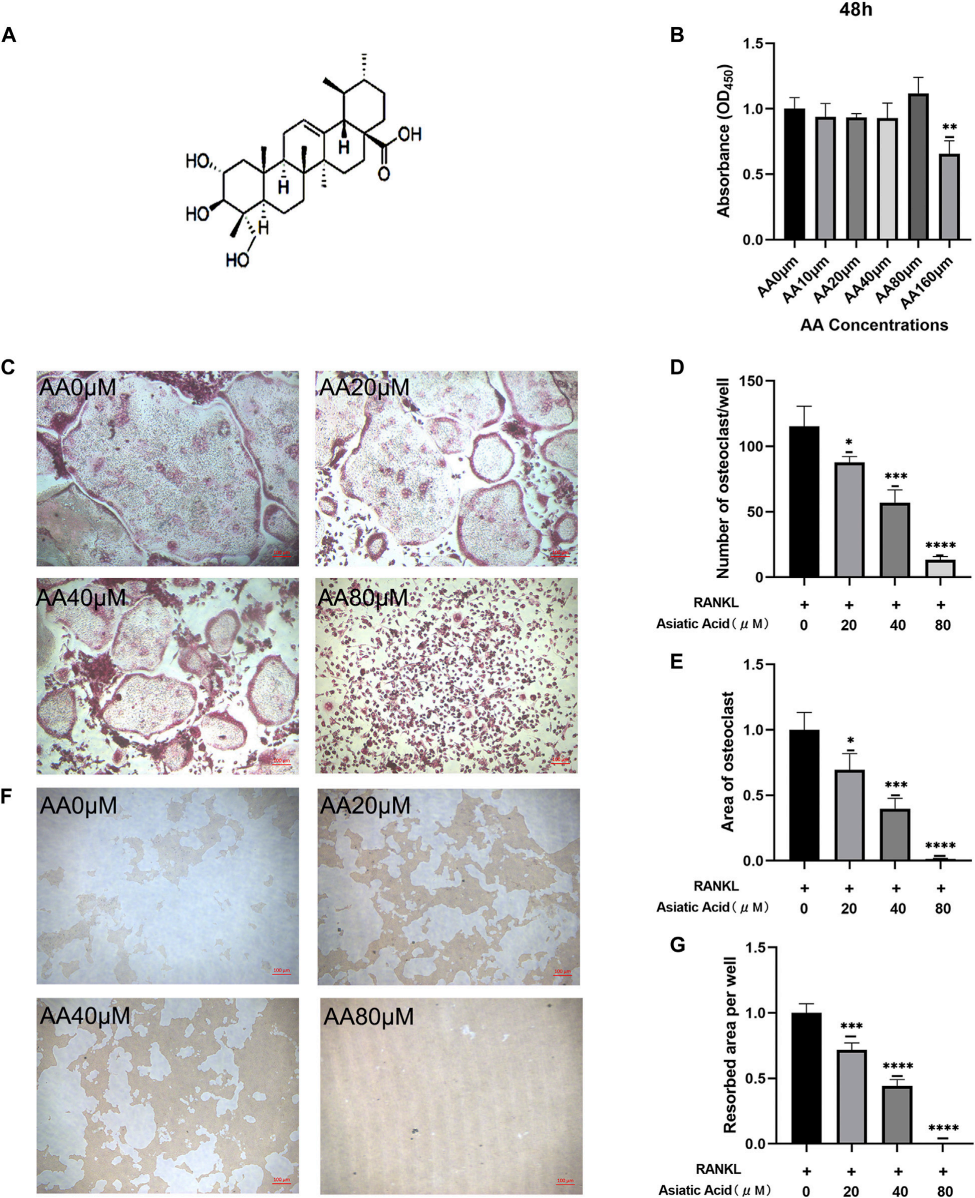
Asiatic acid suppresses the differentiation and bone resorption of osteoclasts. **(A)** The structural formula for asiatic acid. **(B)** Drug toxicity of asiatic acid on BMMs. After BMMs were treated with asiatic acid for 48 h, and the absorbance value of each well was measured with a CCK8 kit (*n* = 4). **(C)** Representative images of asiatic acid inhibition of osteoclast differentiation. BMMs were cultured in *a*-MEM containing 30 ng/mL M-CSF and 50 ng/ml RANKL with or without asiatic acid. After 7 days, the cells were fixed in 4% paraformaldehyde and stained with TRAP (*n* = 3). **(D,E)** Quantitative analysis of the number and area proportion of TRAP-positive osteoclasts (≥3 nuclei) (*n* = 3). **(F)** Representative images of asiatic acid inhibition of bone resorption. **(G)** Quantification analyses of the resorbed area per well (*n* = 3). All values are presented as the mean ± SD (*n* = 3). *p < .05; **p < .01; ***p < .001; ****p < .0001.

The chemical structure formula of asiatic acid is presented in (Figure 1A). First, CCK8 was used to investigate whether asiatic acid was cytotoxic towards the BMMs. Asiatic acid did not exhibit cytotoxicity towards BMMs at concentrations below 80 μM for 48 h (Figure 1B). Therefore, we selected drug concentrations of 20, 40, and 80 μM to study the effects of asiatic acid on osteoclast differentiation and bone resorption. Following exposure to RANKL and MCSF, the effect of asiatic acid on osteoclast differentiation was evaluated by the number and area of TRAP-positive cells. The number and area of TRAP-positive cells were significantly reduced in the group treated with asiatic acid, and asiatic acid inhibited osteoclast differentiation in a dose-dependent manner (Figure 1C−E). Bone resorption experiments were performed to determine the effect of asiatic acid on osteoclast function. The results showed that BMMs in the control group were significantly pitted after induction with RANKL and M-CSF. The area of the pits in the experimental group were significantly reduced following asiatic acid treatment (Figures 1F,G).

### Asiatic Acid Suppresses the Expression of Osteoclast Marker Genes

**FIGURE 2:**
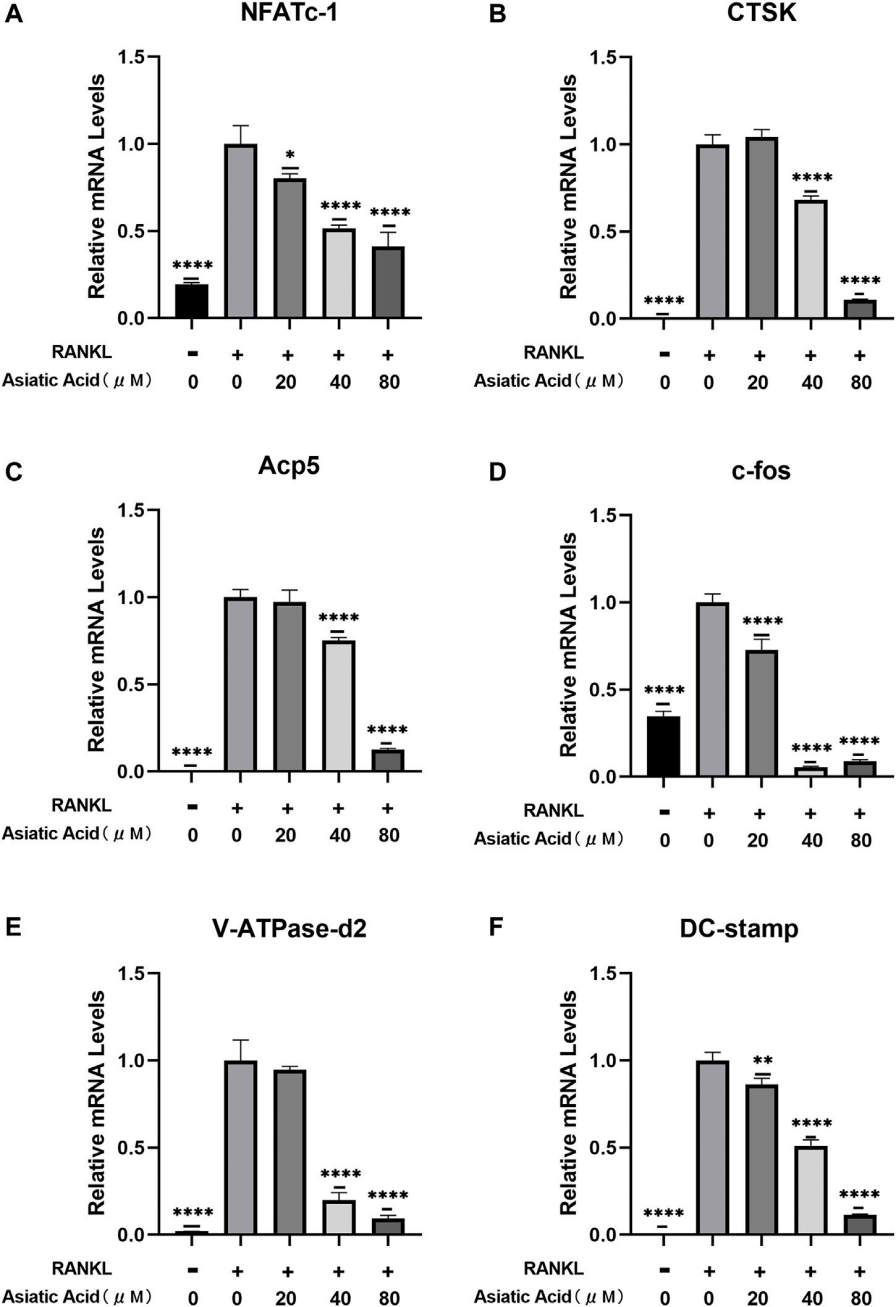
Asiatic acid inhibits RANKL-induced osteoclast-related gene expression. **(A–F)** BMMs were exposed to M-CSF and RANKL for 7 days with or without AA. The expression of osteoclast-specific genes (e.g., NFATc1, c-fos, CTSK, Acp5, DC-stamp, and V-ATpase-D2) were detected with fluorescence quantitative RT-PCR. All values are presented as the mean ± SD (*n* = 3). *p < .05; **p < .01; ***p < .001; ****p < .0001.

To determine the mechanism by which asiatic acid treatment effects osteoclasts *in vitro*, we detected the level of NFATc1, c-fos, CTSK, Acp5, DC-stamp, and V-ATpase-D2 gene expression in osteoclasts using real-time fluorescence quantitative PCR after confirming the inhibition of osteoclast differentiation and bone resorption function by asiatic acid. We found that the aforementioned osteoclast genes were activated under RANKL stimulation. Asiatic acid inhibited the expression of osteoclast-specific genes in a dose-dependent manner (Figures 2A–F). These results suggest that asiatic acid inhibits the function and differentiation of osteoclasts by inhibiting osteoclast-specific gene expression.

### Asiatic Acid Regulates NFATc1 Expression by Inhibiting the NF-κB/MAPK/Akt Signaling Pathway

**FIGURE 3:**
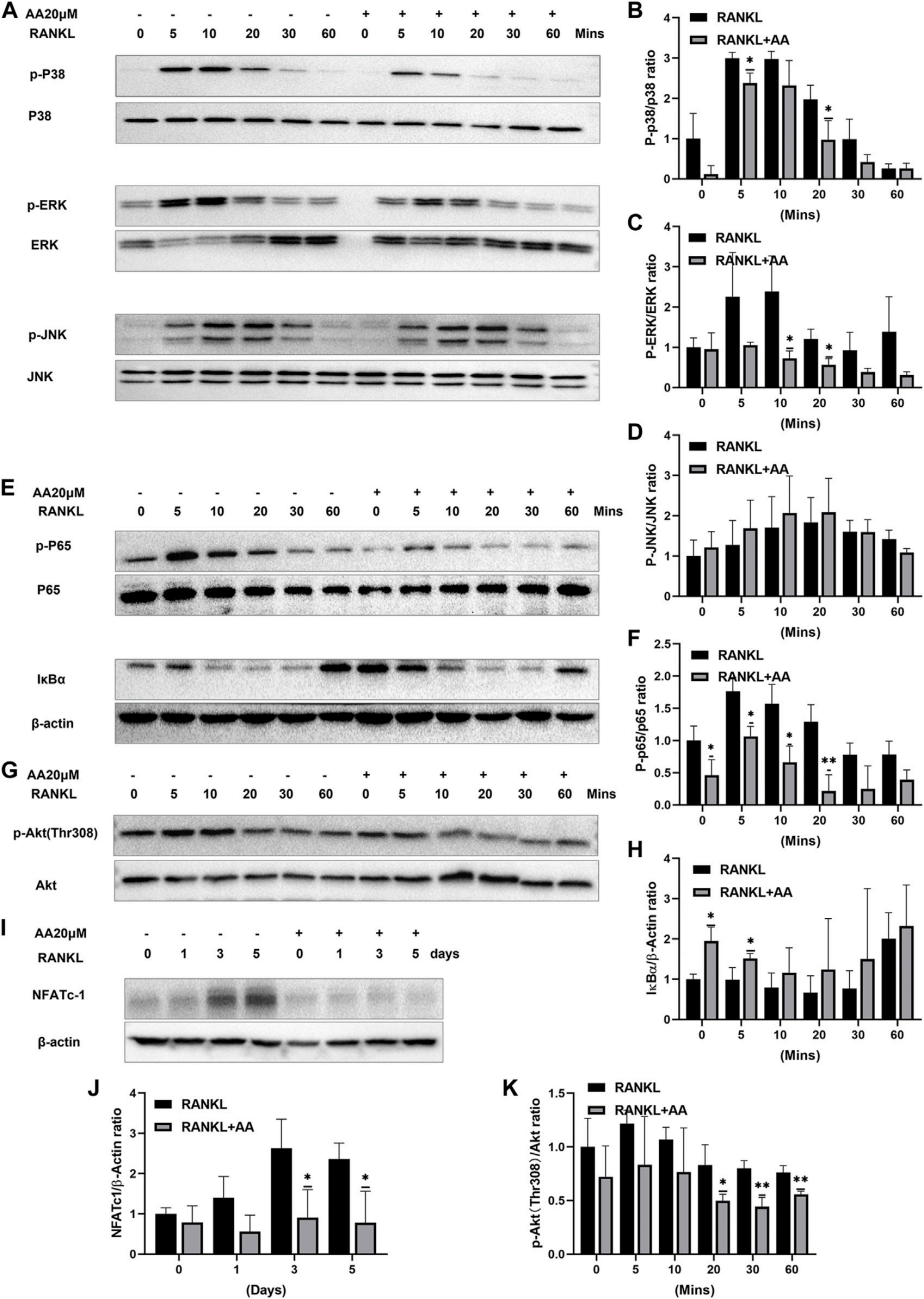
Asiatic acid regulates NFATc1 expression by inhibiting the NF-κB/MAPK/Akt signaling pathway. **(A)** Representative Western Blot images of the effect of asiatic acid on the MAPK pathway. After 2 h of starvation, BMMs were stimulated for 2 h with or without asiatic acid (20 μM). BMMs were stimulated with RANKL for 0, 5, 10, 20, 30, and 60 min **(B–D)** Quantitative analyses of the expression of P-p38, P-ERK, P-JNK, p38, ERK and JNK (*n* = 3). **(E)** Representative Western Blot images of the effect of asiatic acid on the NF-κB pathway. **(F,H)** Quantitative analyses of the expression of P-p65, p65 and IκB*α* (*n* = 3). **(G)** Representative Western Blot images of the effect of asiatic acid on the Akt pathway. **(K)** Quantitative analyses of the expression of P-Akt and Akt (*n* = 3). **(I)** Representative Western Blot images of the effect of asiatic acid on NFATc1. BMMs were stimulated with RANKL for 0, 1, 3, and 5 days in the presence or absence of asiatic acid (20 μM). **(J)** Quantitative analyses of the expression of NFATc1 (*n* = 3). All values are presented as the mean ± SD (*n* = 3). *p < .05; **p < .01 compared with the control group (without AA treatment).

To investigate the molecular mechanism of asiatic acid-mediated inhibition of osteoclasts, we investigated MAPK (e.g., ERK, JNK, and p38), NF-κB, and Akt pathway-related protein expression in RANKL-mediated osteoclastogenesis. In the MAPK signaling pathway, asiatic acid inhibited ERK and p38 phosphorylation but had no significant effect on JNK (Figures 3A–D). We also found that asiatic acid inhibited RANKL-activated p65 phosphorylation and the degradation of NF-κB inhibitory factor IκB*α* (Figures 3E,F,H). These results suggest that asiatic acid inhibited the NF-κB signaling pathway. In addition, since the Akt signaling pathway is also important for osteoclast differentiation, we examined whether Akt activation was inhibited by asiatic acid. It was found that phosphorylation of Akt was decreased following asiatic acid stimulation (Figures 3G,K). Therefore, asiatic acid primarily affects osteoclastogenesis by inhibiting RANKL-induced activation of ERK/p38, as well as the Akt/NF-κB signaling pathway, but not the JNK signaling pathway.

NFATc1 represents an important regulatory factor in osteoclast differentiation. Our previous studies have shown that asiatic acid inhibits the expression of the osteoclast-specific gene, NFATc1; The activation of NFATc1 protein was further explored. Western blotting confirmed that asiatic acid significantly reduced RANKL-induced NFATc1 protein expression (Figures 3I,J).

### Asiatic Acid Could Not Significantly Enhance Osteoblast Differentiation and Mineralization

**FIGURE 4:**
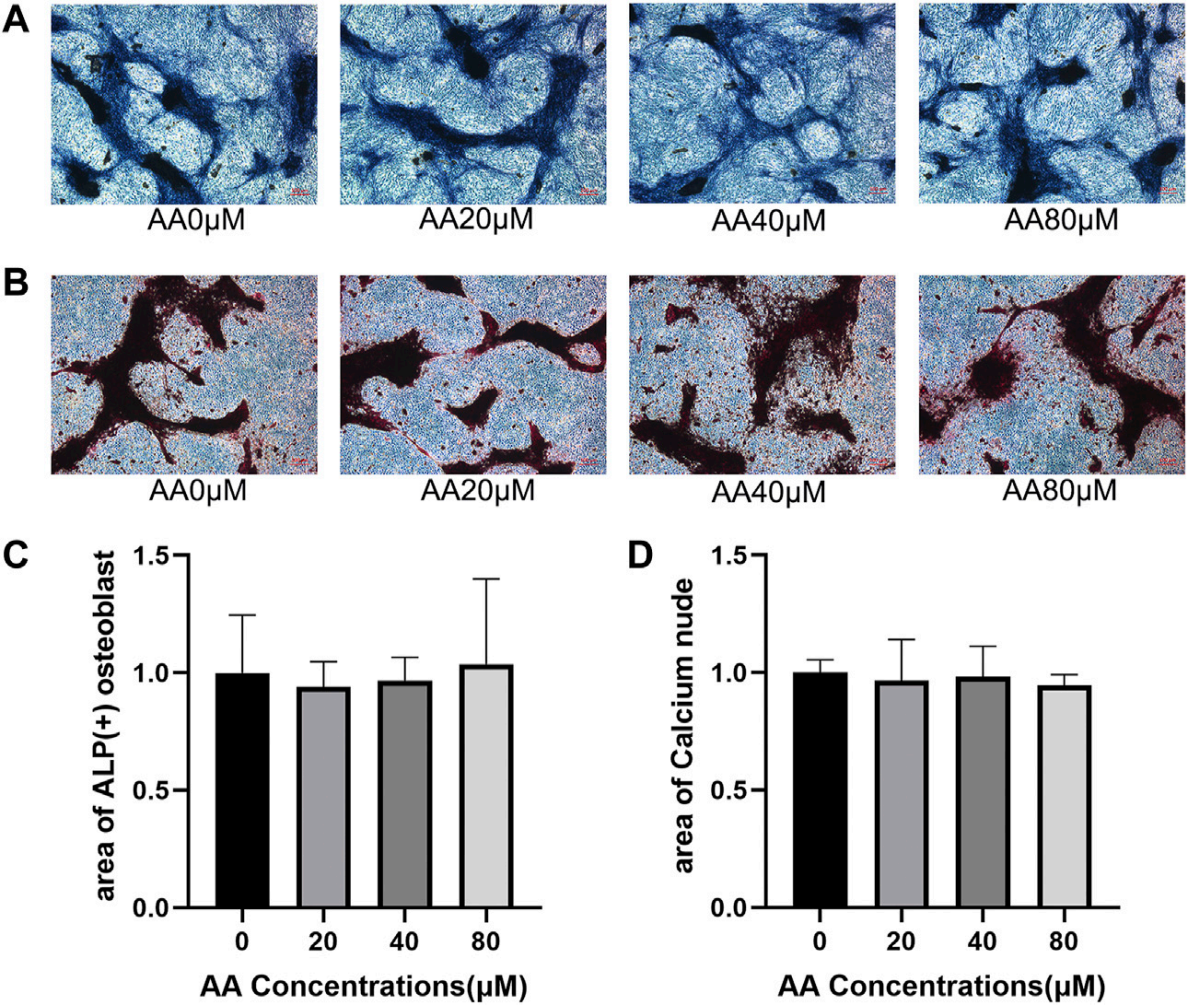
Asiatic acid could not significantly enhance osteoblast differentiation and mineralization. **(A)** Representative images of alkaline phosphatase staining showing that the effect of asiatic acid on osteogenic differentiation. After 7 days of osteogenic induction with or without asiatic acid, osteoblasts were observed by alkaline phosphatase staining kit (*n* = 3). **(B)** Following 21 days of osteogenic induction, mineralized nodules were evaluated by alizarin red staining kit (*n* = 3). **(C)** Quantitative analysis of the percentage of ALP-positive osteoblasts. **(D)** Quantitative analysis of the percentage of mineralized nodules. All values are presented as the mean ± SD (*n* = 3).

Osteohomeostasis is a state of equilibrium that relies on the mutual regulation of osteoblasts and osteoclasts. Since asiatic acid could inhibit osteoclast differentiation, the effect of asiatic acid on osteogenic differentiation and the formation of mineralized nodules was further explored. The effect of asiatic acid on osteogenesis was observed by ALP (Figure 4A) and alizarin red staining (Figure 4B). The results showed that osteoblast differentiation and the formation of mineralized nodules were not significantly affected by asiatic acid (Figures 4C,D).

### Asiatic Acid Suppresses the Progression of Osteoporosis in Ovariectomized Mice

**FIGURE 5:**
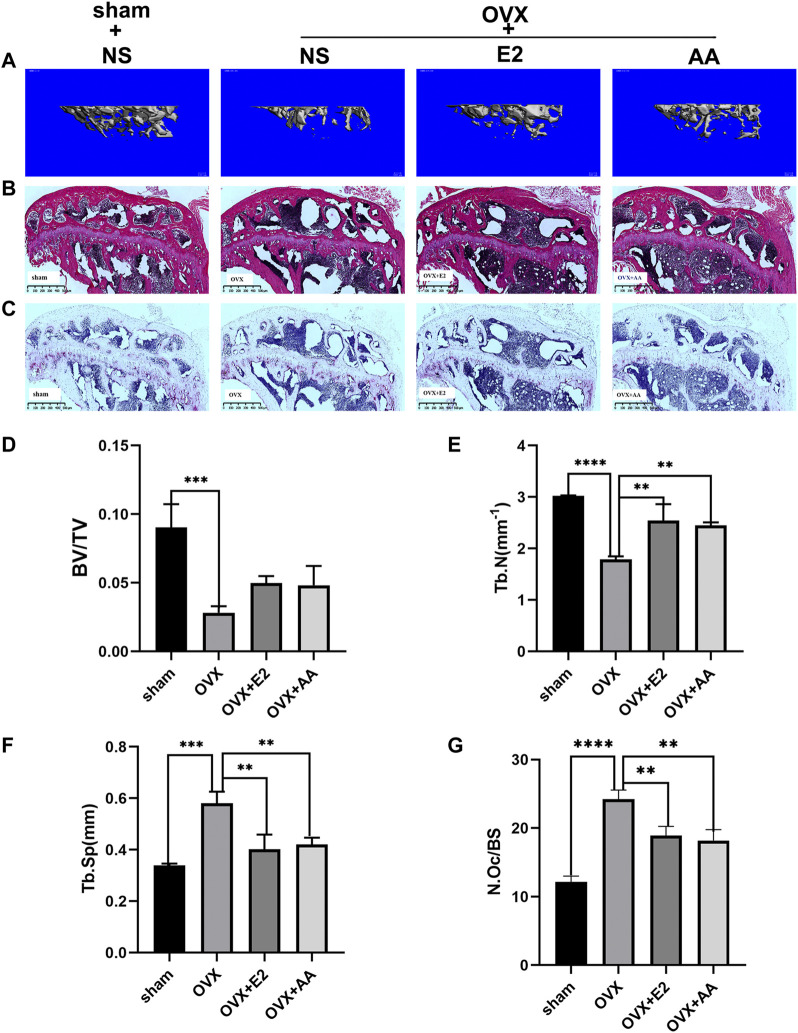
Asiatic acid suppresses the progression of osteoporosis in ovariectomized mice. **(A)** Three-dimensional reconstructed representative image of the mouse tibia. **(B)** Representative images of HE-stained tibia in mice. **(C)** Representative images of Trap staining of mice tibia. **(D–F)** Quantitative analysis of bone volume/tissue volume (BV/TV), bone trabecular number (TB. N), and bone trabecular separation (TB. SP) of mice (*n* = 3). **(G)** Quantitative histological analysis of TRAP-positive cell percentage per bone surface in each group (*n* = 3). All values are presented as the mean ± SD (*n* = 3). *p < .05; **p < .01; ***p < .001; ****p < .0001.

To further verify the effect of asiatic acid on bone loss *in vivo*, an ovariectomy-induced osteoporosis mouse model was used to explore this problem after *in vitro* experiments confirmed the inhibitory effect of asiatic acid on osteoclast differentiation and bone resorption. The mice were randomly divided into four groups: 1) Sham group; 2) OVX group; 3) OVX + estradiol group (OVX + E2); and 4) OVX + asiatic acid group (OVX + AA). The mice were intraperitoneally injected with asiatic acid (10 mg/kg), estradiol (0.15 mg/kg), or normal saline (NS) once every 2 days. After 6 weeks, the mice tibia were collected for a Micro CT scan (Figure 5A) and bone tissue section staining. The results of the related bone parameters showed that compared with the Sham group, the OVX group displayed significant bone loss, which was reflected in decreased bone volume/tissue volume (BV/TV), a reduction in the bone trabecular number (TB.N), and increased bone trabecular separation (TB.SP). Moreover, following asiatic acid and estradiol treatment, the amount of trabecular bone (Tb.N) was increased and there was decreased separation of trabecular bone (TB.SP), indicating a significant improvement in bone loss. However, there was no significant difference in the efficacy between the OVX + asiatic acid group (OVX + AA) and OVX + estradiol group (OVX + E2) (Figure 5D−F). At the same time, the HE (Figure 5B) and TRAP staining (Figure 5C) results in the bone tissue also demonstrated that treatment with asiatic acid could alleviate bone loss in ovariectomized mice. Meanwhile, histological analysis showed that TRAP-positive cell percentage per bone surface decreased significantly in the AA treatment group, when compared with OVX group (Figure 5G).

**FIGURE 6:**
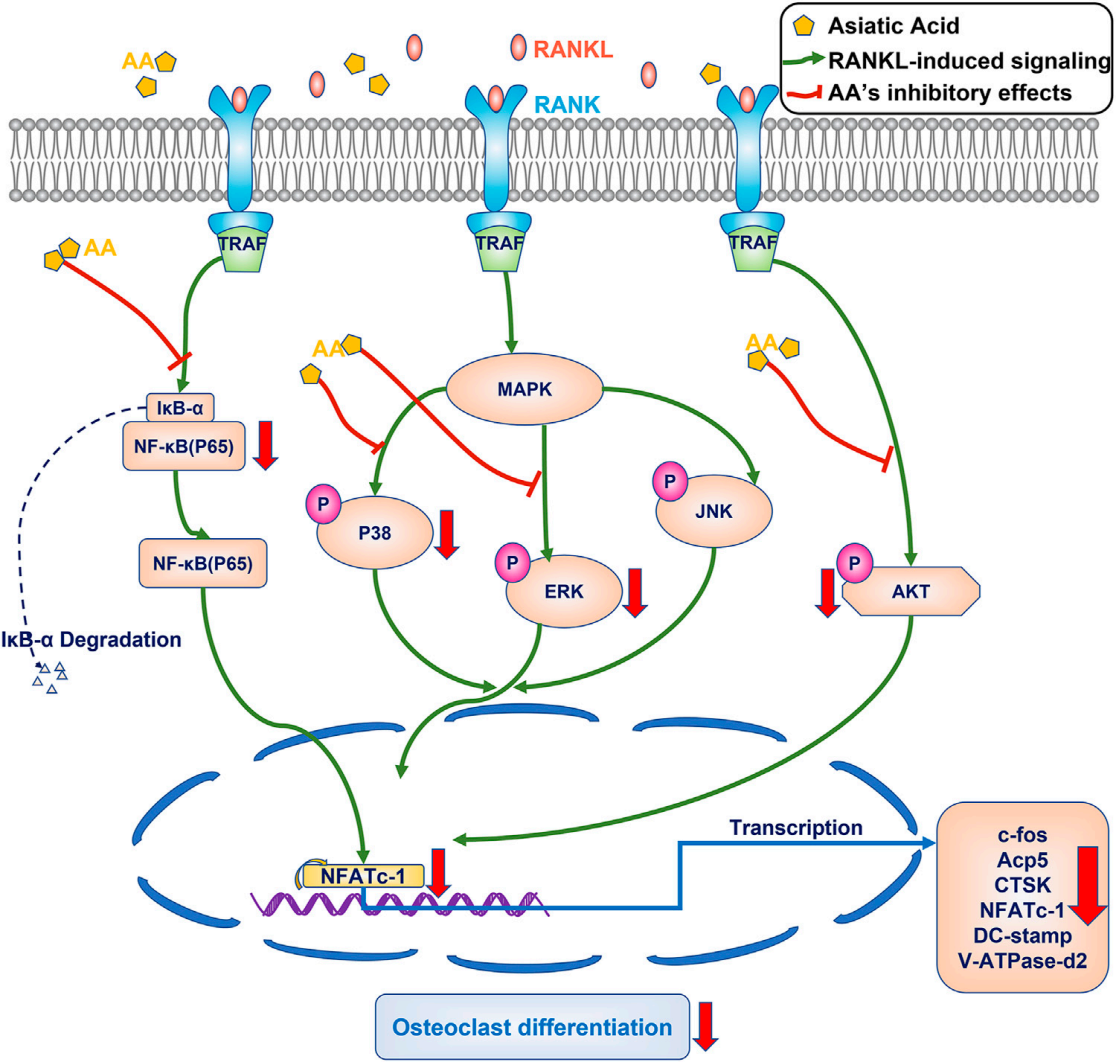
Proposed scheme for mechanism by which asiatic acid inhibits osteoclastogenesis. After binding to RANKL, the RANK can recruit and activate the TRAF6, the NF-κB/MAPK/Akt signaling pathway are activated, resulting in the self-amplification of NFATc1. NFATc1 regulates the expression of osteoclast specific genes, including c-fos, CTSK, Acp5, DC-stamp, and V-ATpase-D2. Our results demonstrate that AA can inhibit osteoclastogenesis by inhibiting the NF-κB/MAPK/Akt signaling pathway.

Asiatic acid attenuates osteoclastogenesis by inhibiting the NF-κB/MAPK/Akt signaling pathway (Figure 6).

## Discussion

The dysregulation of bone homeostasis can cause a range of pathologic conditions (e.g., osteoporosis, rheumatoid arthritis, and bone metastasis of cancer) when osteoclast activity was over-expressed. Thus, the inhibition of osteoclast activity represents the main treatment strategy for the rehabilitation of bone homeostasis. Recently, first-line anti-osteoporosis drugs (e.g., bisphosphonates, estrogens, and denosumab) have shown to reduce the risk of osteoporotic fractures. However, these medications exhibit some side effects, including an increased risk of atypical femoral fractures and osteonecrosis of the jaw (Barasch et al., 2011; Hanley et al., 2017). Thus, novel and appropriate therapeutics are required for the treatment of osteoporosis. In the present study, we showed that asiatic acid could effectively reduce the number of TRAP-positive osteoclasts and decrease the bone resorption area, which was confirmed by the inhibited expression of mature osteoclast-related marker genes. As a molecular mechanism, asiatic acid suppressed RANKL-induced NFATc1 expression by inhibiting the MAPK/NF-κB/Akt signaling pathway. However, asiatic acid exhibited a minimal effect on osteoblast differentiation and mineralization. Furthermore, we found that asiatic acid could alleviate ovariectomy-induced bone loss *in vivo*. In considering the above results, we speculate that asiatic acid attenuates ovariectomy-induced bone loss and osteoclastogenesis *via* the Akt pathway as a novel molecular mechanism.

In general, there are two essential steps that contribute to bone resorption: 1) internal structural changes, which involve rearrangements of the actin cytoskeleton, the formation of sealed compartments between the bone surface and the ruffled border of polarized osteoclasts; and 2) erosion of the underlying bone by exporting hydrogen ions in conjunction with lytic enzymes (Boyle et al., 2003). Moreover, as a biomaterial widely used in bone reconstruction research, hydroxyapatite has a similar effect on the biological activity of osteoclasts and osteoblasts as a mineral found in human bone (MacMillan et al., 2014). Therefore, we used a hydroxyapatite bone plate to study the effect of asiatic acid on bone resorption. In the present study, we demonstrated that multinucleated osteoclast formation was inhibited in a concentration-dependent manner following treatment with asiatic acid. This finding indicated that asiatic acid directly inhibits osteoclast differentiation at a non-toxic dose. Interestingly, alkaline phosphatase and alizarin red staining revealed that asiatic acid did not enhance osteoblast differentiation and mineralization. Furthermore, the results of the bone resorption assay revealed that asiatic acid inhibited the bone resorption activity of mature osteoclasts. These findings indicate that asiatic acid can mitigate osteoclastogenesis in RANKL-treated osteoclasts by suppressing osteoclast differentiation, as well as the bone resorption activity of mature osteoclasts.

The activity of mature osteoclasts is defined by various functional characteristics, of which the three key features include: 1) hydrolase synthesis and directional secretion; 2) acidification of the bone resorption chamber; and 3) effective internalization of the degradation products in the extracellular matrix (Bruzzaniti and Baron, 2006). Moreover, the expression of bone-resorbing genes during the formation of mature osteoclasts are closely related to the functional characteristics of mature osteoclasts (Halleen et al., 2003) (e.g., NFATc1, c-fos, CTSK, Acp5, DC-stamp, and V-ATpase-D2) (Park et al., 2017). NFATc1 is an essential regulator, which induces osteoclast differentiation and functionality. In addition, c-fos can play a synergistic effect with NFATc1 to regulate osteoclast fusion by inducing DC-stamp and V-ATpase-D2 (Lee et al., 2006; Kim et al., 2008). During the process of osteoclast bone resorption, Acp5 is located in cross-cell vesicles, transporting bone matrix degradation products from resorption lacunae to the functional secretion domains of the basement membrane. In addition, ROS produced by Acp5 can destroy organic bone matrix components (Halleen et al., 2003). CTSK represents the primary bone degradation enzyme that is responsible for the degradation of type I collagen in osteoclast-mediated bone resorption (Wilson et al., 2009). Previous research has shown that CTSK is a physiological activator of Acp5 in osteoclasts (Andersson et al., 2003). Thus, these genes were selected to demonstrate the effect of asiatic acid on the functional characteristics of mature osteoclasts. The results of our study show that RANKL activated the expression of osteoclast-specific genes. Asiatic acid had a dose-dependent inhibitory effect on the expression of activated osteoclast-specific genes, which also explained how asiatic acid inhibited the differentiation and bone resorption of osteoclasts.

Osteoclastogenesis is governed by activation of the RANKL-mediated intracellular signal transduction pathway, including NF-κB, MAPK, and Akt. It has been well-established that the NF-κB pathway is a crucial factor involved in RANKL-mediated osteoclastogenesis, which promotes gene transcription when active NF-κB p65 binds to the NFATc1 promoter in the cell nucleus (Xu et al., 2009). The Western blotting results showed that asiatic acid inhibited IκBα degradation and p65 phosphorylation. As a consequence, asiatic acid hindered active p65 translocation into the nucleus; thus, activation of the transcription factor, NFATc1, was also suppressed. In addition, we demonstrated that asiatic acid could considerably suppress RANKL-induced phosphorylation of ERK and p38, whereas a marginal effect was observed in the JNK pathway. Some of our results were in accordance with previous studies, indicating that asiatic acid inhibited IκBα degradation and activation of ERK (Hong et al., 2020). In contrast to the findings of previous studies, the present study found that asiatic acid also inhibited p38 and p65 phosphorylation, which was in accordance with the findings of Jianping Huang’s study (Huang et al., 2019). Similarly, the p38 signalling pathway has been reported to play an important role in osteoclast differentiation and bone remodeling (Matsumoto et al., 2000).

Furthermore, activation of the Akt pathway plays a key role in regulating the differentiation of osteoclasts and mature osteoclast bone resorption. Both Akt1 and Akt2 signaling functions in osteoclast differentiation by positively regulating activation of the NF-κB pathway, whereas the loss of Akt1 and Akt2 protein has been shown to inhibit osteoclastogenesis (Sugatani and Hruska, 2005). The findings of the recent studies indicate that following the phosphorylation of Akt, GSK3β subsequently modulates the expression of the transcription factor, NFATc1 (Moon et al., 2012; Jang et al., 2013). In particular, it has been shown that GSK3β suppresses the capacity of NFATc1 to bind to DNA, which hinders NFATc1-dependent gene transcription (Neal and Clipstone, 2001). Furthermore, the latest research shows that a wide range of cytokines, guanine nucleotide binding protein subunit α13 (G*α*13), 3-phosphoinositide-dependent protein kinase 1 (PDK1), and microtubule actin crosslinking factor 1 (MACF1) can manage osteoclastogenesis by regulating the Akt-GSK3β-NFATc1 signaling axis (Wu et al., 2017a; Lin et al., 2019; Xiao et al., 2020). Our results showed that asiatic acid inhibited Akt phosphorylation and activation of the NFATc1 protein. As a consequence, we believe that asiatic acid inhibited osteoclastogenesis through regulating the Akt-NFATc1 signaling pathway. In accordance with the present results, previous studies have demonstrated that suppression of the Akt signal transduction cascade reduces osteoclastogenesis (Lu et al., 2019; Cui et al., 2020; Yan et al., 2020).

A previous study demonstrated that asiatic acid inhibited osteoclastogenesis (Hong et al., 2020). Different from previous study, our study revealed several important new findings. Our results demonstrated that bone-resorbing genes, such as Acp5 which could destroy organic bone matrix components and transport bone matrix degradation products, DC-stamp which was shown to be critical to regulate osteoclast fusion, were inhibited, indicating that the low expression of bone-resorbing genes was fundamental in the mechanism by which asiatic acid inhibited bone resorption. In addition, our present study found that asiatic acid inhibited p38, p65, Akt phosphorylation during osteoclastogenesis. Therefore, we believed that asiatic acid inhibited osteoclastogenesis through regulating the NF-κB/MAPK/Akt signaling pathway. However, Osteogenic bone formation also plays a key role in bone homeostasis. In our research, we showed that osteoblast differentiation and the formation of mineralized nodules were not significantly affected by asiatic acid. Furthermore, we investigated the degree of osteoprotection of asiatic acid in ovariectomized mice. The osteoprotection effect of asiatic acid *in vivo* consistent with estrogen. Together, these findings provided compelling evidence for asiatic acid as candidates therapeutic against bone loss.

There are some limitations associated with this study. For instance, the precise drug gene target of asiatic acid and more accurate intracellular signal transduction requires further examination. In addition, while we observed that asiatic acid had no significant side effects in the mouse model, further detailed exploration is required.

## Conclusion

In summary, our study clarified the inhibitory effect of asiatic acid on osteoclastogenesis, and further confirmed that asiatic acid can inhibit osteoclastogenesis via the NF-κB/MAPK/Akt signaling pathway. The discovery of a novel signaling pathway involving asiatic acid in osteoclasts provides an important basis for broadening the potential mechanism of asiatic acid in the treatment of osteoclast-related diseases and becoming a potential therapeutic drug.

## Data Availability

The original contributions presented in the study are included in the article/supplementary material, further inquiries can be directed to the corresponding author.

## Abbreviations

AA, Asiatic acid; ALP, Alkaline phosphatase; AKT, Protein kinaseB; BMMs, Bone marrow macrophages; BV/TV, Bone volume to total volume; ERK1/2, Extracellular signal regulated kinase 1 and 2; IκB, Inhibitor of NFκB; IKK, IκB kinase; JNK, C-Jun N-terminal kinase; MAPK, Mitogen-activated protein kinase; MCSF, Macrophage colony-stimulating factor; NFκB, Nuclear factorκB; NFATc1, Nuclerar factor of activated T-cells cytoplasmic 1; OVX, Ovarietomizy; PI3K, Phosphatidylinositol 3-kinase; RANKL, Receptor activator of nuclear factor kappa-B ligand; RANK, Receptor activator of nuclear factor kappa-B; TRAF6, TNF receptor-associated factor 6; TRAP, Tartrate resistant acid phosphatase; Tb.N, Trabecular number; Tb.Sp, Trabecular separation
